# Type IV Secretion System Is Not Involved in Infection Process in Citrus

**DOI:** 10.1155/2014/763575

**Published:** 2014-02-23

**Authors:** Tiago Rinaldi Jacob, Marcelo Luiz de Laia, Leandro Marcio Moreira, Janaína Fernandes Gonçalves, Flavia Maria de Souza Carvalho, Maria Inês Tiraboschi Ferro, Jesus Aparecido Ferro

**Affiliations:** ^1^Faculdade de Ciências Agrárias e Veterinárias de Jaboticabal, Departamento de Tecnologia, Universidade Estadual Paulista (UNESP), 14.884-900 Jaboticabal, SP, Brazil; ^2^Departamento de Engenharia Florestal, Faculdade de Ciências Agrárias, Universidade Federal dos Vales do Jequitinhonha e Mucuri, 39.100-000 Diamantina, MG, Brazil; ^3^Departamento de Ciências Biológicas (DECBI), Instituto de Ciências Exatas e Biológicas (ICEB) and Núcleo de Pesquisas em Ciências Biológicas (NUPEB), Universidade Federal de Ouro Preto, 35.400-000 Ouro Preto, MG, Brazil; ^4^Embrapa Informática Agropecuária, CNPTIA-EMBRAPA, Campinas, SP, Brazil

## Abstract

The type IV secretion system (T4SS) is used by Gram-negative bacteria to translocate protein and DNA substrates across the cell envelope and into target cells. *Xanthomonas citri* subsp. *citri* contains two copies of the T4SS, one in the chromosome and the other is plasmid-encoded. To understand the conditions that induce expression of the T4SS in *Xcc*, we analyzed, *in vitro* and *in planta*, the expression of 18 ORFs from the T4SS and 7 hypothetical flanking genes by RT-qPCR. As a positive control, we also evaluated the expression of 29 ORFs from the type III secretion system (T3SS), since these genes are known to be expressed during plant infection condition, but not necessarily in standard culture medium. From the 29 T3SS genes analyzed by qPCR, only *hrpA* was downregulated at 72 h after inoculation. All genes associated with the T4SS were downregulated on *Citrus* leaves 72 h after inoculation. Our results showed that unlike the T3SS, the T4SS is not induced during the infection process.

## 1. Introduction

*Xanthomonas citri* subsp. *citri* (*Xcc*) is a Gram-negative plant pathogenic bacteria that causes severe disease in many economically important citrus plants [1]. The citrus canker induced by Xcc is a destructive disease characterized by canker lesions on leaves, stems, and fruits; furthermore the pathogen induces defoliation, which results in reduced yield and premature fruit drop [2]. Control is difficult in areas where the disease is already established, and plant eradication is the only effective way to control and prevent the disease spread. Recurrent and severe attacks of the pathogen are responsible for serious economic losses in citrus groves around the world [2].

The focus of the present study is aimed at understanding the role of bacterial secretory systems, specifically the type IV secretory system (T4SS). Seven secretory systems are known and described in prokaryotic organisms, and each one is related to a physiological process [3–5]. Of these, the best studied is the type III secretory system (T3SS), which enables bacterial pathogens to deliver effector proteins into eukaryotic cells [6]. Some bacterial pathogens, including species from *Chlamydia, Xanthomonas, Pseudomonas, Ralstonia, Shigella, Salmonella, Escherichia*, and *Yersinia*, depend on the T3SS to induce damage to the host. In contrast, other organisms including *Agrobacterium tumefaciens, Helicobacter pylori*, and *Legionella pneumophila* depend on the type IV secretory system (T4SS) for virulence induction [7].

The T3SS contains at least twenty distinct proteins, which are subdivided into three parts. The basal body of the system [8–11] is associated with an ATPase and likely facilitates the entry of substrates into the secretion apparatus [12]. This basal body is also associated with a protein needle (the second part of the T3SS), which binds the bacterium to host cells and acts as a conduit for effectors secretion. The third part of the T3SS is composed of three proteins that are exported through the bacterial needle and form a pore on the surface of the host cell, which facilitates the export of toxins into the target cytoplasm [13].

On the other hand, the T4SS is a multiprotein complex that consists of a protein channel (encoded by *virB* and *virD*) through which proteins or protein-DNA complexes can be translocated between bacteria (cell-to-cell communication) and into host cells [14]. Translocation is driven by a number of cytoplasmic ATPases that potentially energize large conformational changes in the translocation complex [15]. There are three functional types of T4SS. The first type, found in many Gram-positive and Gram-negative bacteria and some Archaea, functions in conjugation and in the transfer of T-DNA into plant cells by *A. tumefaciens*. A second type of T4SS mediates DNA uptake in the transformation process (found in *H. pylori*). A third type of T4SS is used to transfer toxic effector proteins or protein complexes into the cytoplasm of host cells (found in *Bordetella pertussis*, *Legionella pneumophila*, *Pseudomonas aeruginosa*, and *Xcc*) [15].

Substrates transported by the T4SS modulate various cellular processes including apoptosis, vesicular traffic, and ubiquitination; furthermore, the number of type IV effector proteins continues to increase [16–19]. However, most of these substrates have not been functionally characterized, and their role in bacterial pathogenesis remains unknown [20].

In many bacteria, these two secretory systems types are well characterized; however, in *Xanthomonas* spp. only the T3SS has been intensively studied [1], in contrast to the limited number of studies involving the T4SS [21, 22]. In Xcc the T4SS deserves more attention, especially since the genome contains two copies of this system (chromosomal and plasmid-borne) [23]. The plasmid copy is homologous to others Xanthomonads that contain the T4SS on extrachromosomal DNA. However, only *Xcc* and *Xanthomonas campestris* contain the chromosomal copy of the T4SS.

To understand the conditions that stimulate expression of T4SS genes in *Xcc* we used qPCR to analyze the *in vitro* and *in planta* expression of 18 ORFs from the T4SS (both chromosomal and plasmid copies) and 7 hypothetical ORFs flanking the T4SS. To validate the qPCR data, we compared expression of 29 ORFs from the T3SS, which are known to be expressed *in planta*.

Our results showed that the T4SS is not induced during the infection process in *Xcc*, but may be very important in cell-to-cell communication.

## 2. Materials and Methods

### 2.1. Microbiological Procedures

*Xcc* strain 306 was previously sequenced by da Silva et al. [23]. *Xcc* was maintained in sterile tap water and grown on nutrient agar (NA) medium containing 3 g beef extract, 5 g peptone, and 15 g agar in 1 L of distilled water at 28°C. After 24 h, three single colonies were transferred to new NA plates and incubated for 12 h at 28°C. These *Xcc* solid cultures were used as pre-inoculum for *in planta* and *in vitro* studies. For *in planta* studies, pre-inoculum cultures were aseptically transferred to sterile flasks containing 50 mL of nutrient broth (NB) and the bacterial concentration was adjusted to 10^8^ CFU/mL (OD_600_ = 0.3). This bacterial suspension was taken up in a 1 mL needleless syringe and used to infiltrate orange leaves (*Citrus sinensis* cv. pera) grown in 20 L capacity pots. A total of nine plants were inoculated (20 leaves per plant) and maintained for 72 h in a growth room at 28°C, with a 12 h photoperiod and light intensity ~2,000 lux. Three plants were used for each incubation period. After multiplication, inoculated leaves were collected, sliced into thin strips with a razor blade, and placed in a beakers with sterile distilled water in an ice bath with gentle agitation. Samples from each plant were placed in separate beakers for bacterial exudation. After 5 min, leaf debris was removed by filtration through gauze, and bacterial cells were recovered by centrifugation at 5,000 ×g for 5 min at 4°C. Total RNA was extracted immediately, as described below. For *in vitro* studies, 1 mL of each *Xcc* suspension at 10^8^ CFU/mL were aseptically transferred to three Erlenmeyer flasks and incubated for 12 h at 28°C with shaking (200 rpm). After incubation, bacterial cells were harvested by centrifugation at 5,000 ×g for 5 min. Total RNA was extracted as described below. Thus, we obtained three independent biological replicates for *Xcc *growth *in vitro* and *in planta*. All cultivation media were obtained from Difco Chemical Co., Detroit, USA. For confirmatory RT-qPCR, the same procedures were followed except the plant incubation period was only 72 h, due to RNA quality and concentration sufficient to yield reliable results. The time point 72 hours was the shortest period to allow obtaining satisfactory bacterial mass to the proposed analyzes involving gene expression. The bacterial cells concentration in short time (12 or 24 hours) don't allows this study. For this reason, some works are using culture media that mimic vegetable conditions to investigate the infectious process under these time scales [24, 25].

### 2.2. Extraction of RNA from *Xcc*

Each inoculated flask represented one independent biological replicate. RNA was extracted using an Illustra-RNAspin Mini RNA Isolation Kit (Amersham Biosciences) following the manufacturer's instructions. To ensure that samples did not contain DNA, PCR was performed using RNA samples treated with DNase I as a template. PCR conditions consisted of an initial denaturing step of 94°C for 3 min, followed by 35 cycles of a denaturation at 94°C for 30 s, annealing at 60°C for 30 s, and elongation at 72°C for 2 min. A final elongation step of 72°C for 4 min was performed, and then samples were maintained at 4°C until needed. The amplification reaction was conducted in a total volume of 25 *μ*L containing 200 ng RNA in 2.5 *μ*L, PCR buffer (2.5 *μ*L, Invitrogen), 1.5 mM MgCl_2_, 0.2 mM dNTP, 300 nM 16S rRNA primer [26], and 1 U Taq DNA polymerase (Invitrogen). The products were electrophoresed in a 1% agarose gel with TAE buffer, stained with ethidium bromide, and visualized using a UV transilluminator. No products were observed (data not shown). To verify the quality of extracted RNA, the samples were analyzed by electrophoresis in a 1% agarose gel using TAE buffer followed by ethidium bromide staining. The A260/280 ratio of RNA samples was measured, and RNA was quantified using a NanoDrop ND-1000 spectrophotometer (NanoDrop Technologies, Wilmington, DE, USA). RNA samples were stored at −80°C until needed.

### 2.3. Gene Expression Using RT-qPCR

First strand cDNA synthesis and all RT-qPCR reactions were done using the SuperScript III First-Strand Synthesis SuperMix for RT-qPCR (Invitrogen) as recommended by the manufacturer's specifications except for the amount of cDNA in each reaction, which was 20 ng. All PCR was performed with SYBR Green on a 7500 Real-Time PCR instrument (Applied Biosystems) using three biological replicates and three technical replicates (one for each biological replicate). PCR was for 2 min at 50°C, 10 min at 95°C, followed by 40 cycles of 15 s at 95°C, and 1 min at 60°C. To determine PCR efficiency, standard curves were generated using cDNA samples at five dilutions and measured in triplicate. *rpoB, atpD*, and *gyrB* were used as reference genes in all experiments [26]. To perform relative expression analysis, we used the 2^−ΔΔ^ CT method [27]. Primer features are presented in Table 1.

## 3. Results and Discussion

### 3.1. qPCR Results for the T3SS

**Figure 1:**
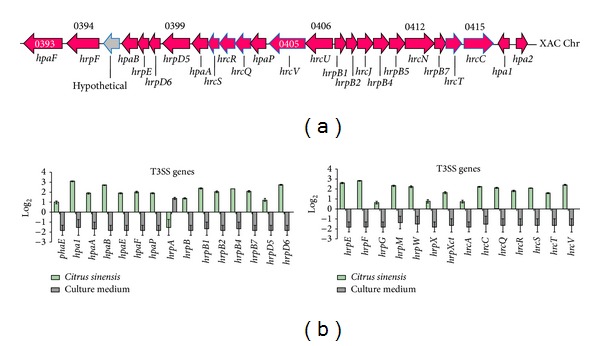
T3SS genes in *Xanthomonas citri* subsp. *citri* and their expression levels. Schematic representation of T3SS genes in *Xanthomonas citri* subsp. *citri* and their expression levels. (a) shows functional maps of T3SS genes on the chromosome. The expression levels of genes in the T3SS (b) are indicated. Solid green bars indicate *in vivo* expression with respect to their expression *in vitro* (black bars). Error bars indicate standard deviation of the replicates.

The results obtained from *Xcc* growing *in planta* or *in vitro* are presented in Figure 1. From the 29 T3SS genes analyzed by qPCR, only *hrpA* was downregulated at 72 h after inoculation (Figure 1(b)). *hpa1*, *hpaB*, *hrpD6*, *hrpE*, and *hrpF* showed the highest rates of expression, whereas *phaE*, *hrpG*, *hrpX*, and *hrcA* showed the lowest levels of expression. Interestingly, *hrpB4 *and *hrpXct*, which were shown to be necessary for *Xcc* pathogenesis [28], were not among the most upregulated genes in this study.

### 3.2. qPCR Results for the T4SS

**Figure 2:**
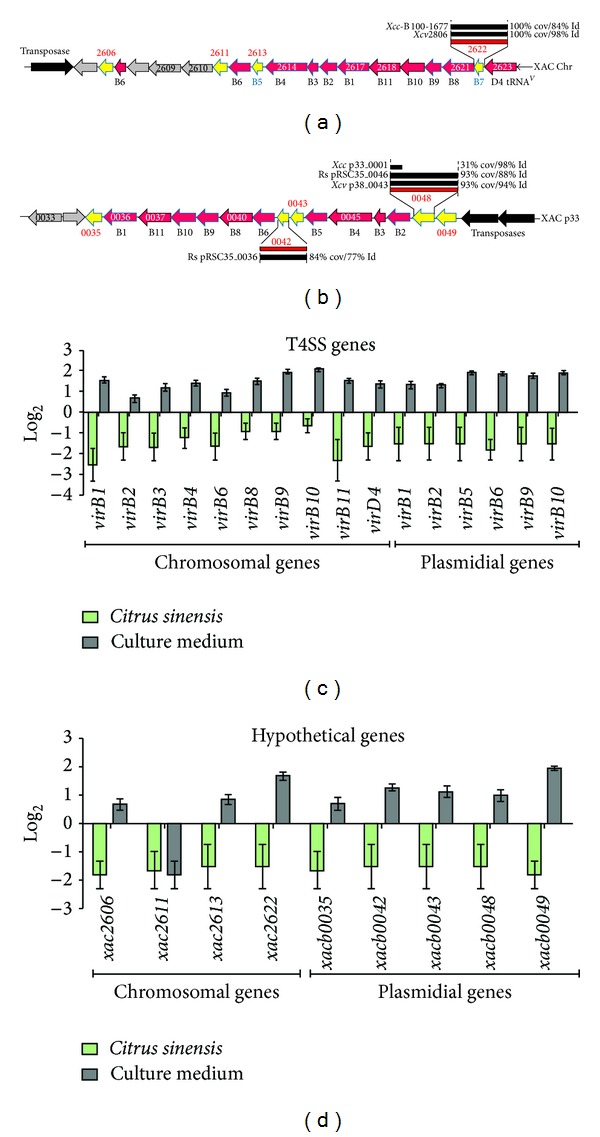
T4SS genes in *Xanthomonas citri* subsp. *citri* and their expression levels. Schematic representation of T4SS genes in *Xanthomonas citri* subsp. *citri* and their expression levels. (a) and (b) show functional maps of T4SS genes on the chromosome (a) and plasmid pXAC64 (b), respectively. The expression levels of genes in the T4SS (c) and ORFs localized near the *virB* genes (d) are indicated. Solid green bars indicate *in vivo* expression with respect to their expression *in vitro* (black bars). Error bars indicate standard deviation of the replicates.

The results for the expression levels of T4SS genes *in planta* and *in vitro* are shown in Figure 2. All genes associated with the T4SS were downregulated on *Citrus* leaves 72 h after inoculation (Figure 2(c)). We also analyzed the expression of hypothetical genes located near *virB* in chromosomal DNA (XAC2606, XAC2611, XAC2613, and XAC2622) and in plasmid pXAC64 (XACb0035, XACb0042, XACb0043, XACb0048, and XACb0049) (Figure 2(d) and Table 1). ORFs representing hypothetical genes in both the chromosome and plasmid were down-regulated when *Xcc* was cultivated in *Citrus sinensis *for 72 h. An exception was XAC2611, where expression was similar when *Xcc* was cultivated in culture medium or *in planta* (Figure 2(d)).

Our results clearly show different gene expression profiles for the T3SS and T4SS in *Xcc* during *Citrus* infection. The T4SS was previously described in *A. tumefaciens*, where it mediates the transfer of DNA and protein substrates to plants and other organisms via a cell contact-dependent mechanism [14]. In *Xcc*, the genome sequence revealed the presence of two *virB* operons, one on the chromosome and a second copy in the 64 kb plasmid pXAC64 [23]. The chromosomal genes *virB1*, *virB3*, *virB4*, *virB8*, *virB9*, and *virB11* and the plasmid-encoded genes *virB1*, *virB2*, *virB4*, *virB6*, *virB9*, and *virB11* were all down-regulated *in planta* (Figure 2(c)). This was somewhat surprising because the T4SS is essential for a successful infection in many Gram-negative pathogenic bacteria [29]. The fact that ORFs representing hypothetical genes in both the chromosome and plasmid near the *virB* genes were also down-regulated under the same condition (Figure 2(d)) indicates that they could be related to the T4SS system in *Xcc*. Recent studies in *Brucella suis* [30] and in *A. tumefaciens* [31] showed that alterations in *VirB8* result in protein dimerization, a process that modifies the T4SS structure and affects bacterial virulence. In *Streptococcus suis* [32], the knockout of the virD4-89K and virB4-89K of the T4SS eliminated the lethality of a highly virulent strain and impaired its ability to trigger host immune responses in mice. Recent studies continue to demonstrate the importance of the T4SS and its components in virulence; thus the lack of induction of Xcc T4SS genes in the present study is intriguing. However, in *Xanthomonas campestris* pv. *campestris* T4SS-deletion mutant displayed the same virulence as wild type and authors conclude that T4SS is not involved in pathogenicity in that *Xanthomonas* [33].

Wang et al. [34] presented evidence that *in planta* transfer of a 37 kb plasmid (pXcB) from *Xanthomonas aurantifolii* to *Xanthomonas citri* can occur via T4SS. Thus, at least the T4SS copy present in *Xcc* plasmid can play a hole in horizontal gene transfer.

All but one of the twenty-nine genes from the T3SS of *Xcc* were up-regulated *in planta*. Laia et al. [28] showed that *Xcc* mutants containing mutations in *hrpB4* or *hrpXct* failed to cause disease and growth in citrus leaves was lower than the wild-type *Xcc* strain 306. These two genes, which are not among the most up-regulated genes in the present study, are part of the *hrp* (hypersensitive reaction and pathogenicity) system and comprise part of the T3SS [35]. In the related pathogen, *Xanthomonas campestris* pv. *vesicatoria* (*Xcv*), a *hrpB4* mutant was unable to cause disease in susceptible pepper plants or the hypersensitive reaction in pepper plants carrying the respective compatible R gene [36]. Previously, *hrpXv* was shown to be necessary for transcriptional activation of five *hrp* genes (loci *hrpB* to *hrpF*) [37], and *hrpB4* was required for complete functionality of the T3SS in *Xcv* [36]. Thus it is apparent that a gene does not have to be strongly up-regulated during infection to play an important role in virulence. Multiple genes, including *hpaA*, *hpaE*, *hpaF*, *hpaP*, *hrpB, hrpB1*, *hrpB2*, *hrpB7*, *hrpD5*, *hrpM*, *hrpW*, *hrcC*, *hrcQ*, *hrcS*, *hrcT,* and *hrcV*, were expressed at levels similar to *hrpXct* and *hrpB4* and are good candidates for further studies into their roles in citrus canker disease.

Among the most up-regulated genes, *hrpD6* warrants further attention. A *hrpD6* mutant of *Xanthomonas oryzae* pv. *oryzae* (*Xoo*) failed to trigger a hypersensitive response in tobacco and was nonpathogenic in rice because the mutation in *hrpD6* impacts the secretion of T3SS effectors, such as Hpa1, which is translocated through the T3SS [38]. Our qPCR data showed that *hpa1* was the most up-regulated gene during *Citrus sinensis* infection by *Xcc*, followed by *hrpD6* (Figure 1(b)). Conversely, *hrpA*, which encodes an important component of the T3SS pilus [39], was down-regulated in our study. Previously, *hrpA* mutants of *Pseudomonas syringae* pv. *tomato* DC3000 showed reduced accumulation of structural components of the *hrp* pilus [39]. Haapalainen et al. [40] showed that soluble plant cell signals induce the expression of *hrp/hrc* genes and specifically upregulate *hrpA*. Interestingly, they found that HrpA does not accumulate to high levels intracellularly, suggesting that some kind of feedback regulation takes place between secretion and production of HrpA, perhaps through degradation of intracellular HrpA. Our work shows that this may also be applicable to *hrpA* gene in Xcc: its mRNA may be degraded faster, just as happen with HrpA in *Pseudomonas syringae*.

## 4. Conclusions

Our finds confirm that the T3SS genes are induced in the successful infection of *Citrus sinensis* by *Xcc*, and therefore are excellent targets to help control or decrease the severity of citrus canker, whereas T4SS seems not have any relation with virulence. On the other hand, our results show that T4SS is not induced under the same infection conditions, which could indicate that it is not necessary for infection but only for cell-to-cell communication.

## Figures and Tables

**Table 1:** General features of the T3SS and T4SS primer sequences.

XAC ID	Gene	Gene ID	Product (NCBI Annotation)	Primers (Forward)	Primers (Reverse)	Amplicon (bp)	System
XAC0293	*hrpB *	1154364	ATP-dependent RNA helicase	GCCGTATCCCGTTGACCTT	ACCGCCCCTTCATCTCTTTT	81	T3SS
XAC0393	*hpaF *	1154464	HpaF protein	GGCGAGCGTTTTGACAA	CCGGAGATCCTGGCAGTTT	57	T3SS
XAC0394	*hrpF *	1154465	HrpF protein	GCCGCGACGCAGTTG	CGGTGATCGAGTTGTTCAA	62	T3SS
XAC0396	*hpaB *	1154467	HpaB protein	GTCCGTGTACGCGCAAGAC	TGACGCCGGCATCATTG	53	T3SS
XAC0397	*hrpE *	1154468	HrpE protein	ACGAGGCCCAGAAGTCCAT	CCACGTTGAAGTCCAGATCATTT	66	T3SS
XAC0398	*hrpD6 *	1154469	HrpD6 protein	ACCGATACGGTCACCCAAGA	GATCCGCCGCCTTGGT	55	T3SS
XAC0399	*hrpD5 *	1154470	HrpD5 protein	GCCCTGAAGTGCGTTCGTTA	TCACGGACGGGTCTTGCT	54	T3SS
XAC0400	*hpaA *	1154471	HpaA protein	CGCCACCCCAACAGAAAA	ACGGCATCAGCTCCATCAA	59	T3SS
XAC0401	*hrcS *	1154472	HrcS protein	CGACGATCTAGTGCGATTTACCT	CGACCACCGGCAAGGA	68	T3SS
XAC0402	*hrcR *	1154473	Protein T3SS	ACACGGGAACGCGAAAAA	CCTTGGGCCAGATCTGTTGT	58	T3SS
XAC0403	*hrcQ *	1154474	HrcQ protein	GCAGCTGGAAGTGGACCAA	GGCTGCAAACCCGACAAC	57	T3SS
XAC0404	*hpaP *	1154475	HpaP protein	TGTCGATGGCACGAAATACC	CCCAACCGCGCCATT	58	T3SS
XAC0405	*hrcV *	1154476	HrcV protein	GCCGCAAGTTGGTCGAGTT	CTCTGCCATATCGCGATTCC	58	T3SS
XAC0406	*hrcU *	1154477	HrcU protein T3SS	AGCCGCGGTGCTCTTTG	TAGTCGCCGGCCGATTT	51	T3SS
XAC0407	*hrpB1 *	1154478	HrpB1 protein	CTGCGGCCAGAGCTGAAG	ACCGCGCTTGATGGAAATC	60	T3SS
XAC0408	*hrpB2 *	1154479	HrpB2 protein	CAGCGCAGCAGATCAAGTTG	CGCCGACGCTGACATTG	69	T3SS
XAC0409	*hrcJ *	1154480	HrcJ protein	GAGCGGCACGCCAATC	TGTCGAGATCAAGCCATCCTT	54	T3SS
XAC0410	*hrpB4 *	1154481	HrpB4 protein	GGACAACACGCGGATCGA	GTCCGTCCAGCGCTGAA	52	T3SS
XAC0411	*hrpB5 *	1154482	Protein T3SS HrpB	CGCACGCCTGGGATATG	AGGCCGGATTCGTTCCA	63	T3SS
XAC0412	*hrcN *	1154483	T3SS ATPase protein	TCGATGGGCACCTGATTCTC	CGTCGATTGCCGGGTACT	63	T3SS
XAC0413	*hrpB7 *	1154484	HrpB7 protein	CCGCGCTGATGGACAAG	GCTGCAGGATCGTGTCGATA	60	T3SS
XAC0414	*hrcT *	1154485	HrcT protein	ATCCAGCAATCCGACAGCAT	CACCGGTGCGGACAGTTT	54	T3SS
XAC0415	*hrcC *	1154486	HrcC protein	CAGATGCGGCTGGATGTG	CCGTCCACGGTGTTGGAT	59	T3SS
XAC0416	*hpa1 *	1154487	Hpa1 protein	GCAGCAGGCCGGTCAGT	TTCATCAGCATCTGGGTGTATTG	57	T3SS
XAC0459	*phaE *	1154530	monovalent cation/H + antiporter subunit E	CCATCCCGGCTTCATCTG	GCGATGCCGTGAATGTTG	54	T3SS
XAC0618	*hrpM *	1154689	Glucosyltransferase MdoH	CAAGGGCCTGCACTGGAT	GCATCAACAGACCCCACATC	86	T3SS
XAC1265	*hrpG *	1155336	HrpG protein	CGGCAAGCCGATCGTATT	GAAAAGAGCAGCCAGGCAAT	54	T3SS
XAC1266	*hrpXct *	1155337	HrpX protein	GCCTACAGCTACATGATCACCAAT	TGCGGCCACTTCGTTGA	63	T3SS
XAC1520	*hrcA *	1155591	Heat-inducible transcriptional repressor	AGTTCGCGTTTCGGCATATC	CGGTTCTGCACCTCGTTGT	91	T3SS
XAC1994	*hrpX *	1156064	HrpX-like protein	CTGGTCTGATGCGAGCTTTCT	GCGTACTCGTCCACGATCAA	90	T3SS
XAC2047	*phaE *	1156118	PHA synthase subunit	AGCAGGGTGCATCGAAGAAG	TTTTCGGAGCCGCCTTTT	63	T3SS
XAC2606	—	1156677	Hypothetical protein	CCCTCAGTGTGGGCCAGAT	AGGAGGCGGTTGAGGGATA	58	H_T4SS_C
XAC2611	—	1156682	Hypothetical protein	GGGAGCCCGCAATGTG	GGAGGCGTGGGAATATCATCT	59	H_T4SS_C
XAC2612	*virB6 *	1156683	VirB6 protein	TGCAGCAGGGAGGATTAGGA	CGGCGGTGCGGTGAT	56	T4SS_C
XAC2613	—	1156684	Hypothetical protein	CGAAGGGAGATGGAAGCAGTT	GCTCAACAGATCGGCTGGAA	59	H_T4SS_C
XAC2614	*virB4 *	1156685	VirB4 protein	GACGTCGCTTGGTGGAGAAA	TTCCTCGGCGCGAATCT	53	T4SS_C
XAC2615	*virB3 *	1156686	VirB3 protein	GCCCCGCCATGTTTTTG	CGCCCGCACCAATGAA	54	T4SS_C
XAC2616	*virB2 *	1156687	VirB2 protein	TCGCCGATGCCAAAATG	CAGCAACGAAATGAATAGCAATG	62	T4SS_C
XAC2617	*virB1 *	1156688	VirB1 protein	CGTATCGAGTCGTCGCGTAAT	CACCAGGCGTCCACCAA	57	T4SS_C
XAC2618	*virB11 *	1156689	VirB11 protein	CGCTGGTCAACCACATTCC	CCTGGCGTCCTCAATCGT	56	T4SS_C
XAC2619	*virB10 *	1156690	VirB10 protein	CCATTGCAGCTCTCTTGATTCTC	GAATCCTCCCCGCTTTGC	61	T4SS_C
XAC2620	*virB9 *	1156691	VirB9 protein	GCTGAGCCCGAACGAAAAA	GCTCCCATCCACCAGTGAA	59	T4SS_C
XAC2621	*virB8 *	1156692	VirB8 protein	TCGTCATGGCAGATGCGTAT	CCGAAGTTGGGCTCAAGCT	61	T4SS_C
XAC2622	—	1156693	Hypothetical protein	GGCGGATTCCAATATGCAGTT	TGGCCCGATCAAGGTGTAG	59	H_T4SS_C
XAC2623	*virD4 *	1156694	VirD4 protein	TCGCTGGAATCCATTGACCTA	TCAAATCCGAAACGCGAAAT	59	T4SS_C
XAC2922	*hrpW *	1156993	HrpW protein	GGCGGACACACCGACATC	TGCCTTTCAGGGTGGAGTCTT	58	T3SS
XAC3122	*hrpA *	1157193	ATP-dependent RNA helicase	TGCGCGCTCTGAATTCG	GGACTGGGTAAGATCTTCATGCA	76	T3SS
XACb0035	—	1158522	Hypothetical protein	CGAGACGAGAACGGCCATT	TGTCACGCAGCAATTCGAA	55	H_T4SS_P
XACb0036	*virB1 *	1158523	VirB1 protein	CGTTACAACCTCGCCAAATATG	CTGCCCGCCTTGAGATTG	74	T4SS_P
XACb0037	*virB11 *	1158524	VirB11 protein	CCCGGAAGCGGTTGAGT	CGTGAGCGACCCCTTGTG	58	T4SS_P
XACb0038	*virB10 *	1158525	VirB10 protein	TCTACGTGAACAAAGACCTCGATT	CGCTTGGCTCCGTCGAT	64	T4SS_P
XACb0039	*virB9 *	1158526	VirB9 protein	GCTCACGCGGCGAAGTT	GCACTTGCTTGACACGATCATC	58	T4SS_P
XACb0040	*virB8 *	1158527	VirB8 protein	CTCGAACAAGCTCAAACAGGAA	AACAGCAGCAACCCGATGA	60	T4SS_P
XACb0041	*virB6 *	1158528	VirB6 protein	GCGCAGCTCGTCATCGT	CGCGGACACCCAACATG	55	T4SS_P
XACb0042	—	1158529	Hypothetical protein	CCGGGAACCCGCTTTTA	GGAAGCCACTGCCGAAACT	52	H_T4SS_P
XACb0043	—	1158530	Hypothetical protein	TTCGCGGCCCTCATCTC	AGGGCGACTTGTTGAACTTGTT	58	H_T4SS_P
XACb0044	*virB5 *	1158531	VirB5 protein	CAGGCCGCCTACGAGAAG	GGGATTGGCGAACAGGTCTT	57	T4SS_P
XACb0045	*virB4 *	1158532	VirB4 protein	GGAACAGGCAAAAGACGTCAAC	CCCGACAAGGTTTGAACGA	57	T4SS_P
XACb0046	*virB3 *	1158533	VirB3 protein	TCTTGCGATGAGCTTTTATTTCC	CCCAAAGTGATCGCGTGATT	59	T4SS_P
XACb0047	*virB2 *	1158534	VirB2 protein	CGATCTGGCGGCTAACGT	ATGCGAGCTGCGGAAGAA	56	T4SS_P
XACb0048	—	1158535	Hypothetical protein	GGCGGATGACGTTGAAGCT	CGCGGTAACGACCTCATAAAC	56	H_T4SS_P
XACb0049	—	1158536	Hypothetical protein	ACCACGGATCCTGGCAAAT	AGCAGCCCCCGTGAACA	51	H_T4SS_P

This table provides primers sequences, ORF's identification ID, gene's name, and respective *Xanthomonas citri* subsp. *citri* secretory systems. T4SS: type IV secretion system; T3SS: type III secretion system; C: chromosomal copy; P: plasmid copy; H: hypothetical genes associated with the T4SS.
